# Monoclonal Gammopathy With Masked IgG Kappa Deposits in a Patient With Systemic Lupus Erythematosus: A Case Report

**DOI:** 10.7759/cureus.88469

**Published:** 2025-07-21

**Authors:** Marshall J Weber, Rashi Agrawal, William Bedinger, Kristianna Singh, Alyce M Oliver, Hong Shi

**Affiliations:** 1 Department of Internal Medicine, Augusta University Medical College of Georgia, Augusta, USA; 2 Department of Pediatrics, Augusta University Medical College of Georgia, Augusta, USA

**Keywords:** autoimmune disease, immunofluorescence, kidney biopsy, membranous-like glomerulopathy, systemic lupus erythematosus

## Abstract

We present the case of an 18-year-old female with a recent influenza B infection, anemia, fatigue, arthralgias, obesity, and a new diagnosis of systemic lupus erythematosus. Initial evaluation revealed proteinuria suggestive of renal involvement. Kidney biopsy confirmed membranous glomerulonephritis with masked IgG deposits (MGMID), despite negative paraprotein studies and lack of classic immunofluorescence staining. Treatment with mycophenolate mofetil led to significantly improved proteinuria without corticosteroids. This unique case underscores the importance of IF-P in patients with unclear renal histopathology and adds to the limited literature on MGMID occurring in the context of well-defined autoimmune disease.

## Introduction

Membranous-like glomerulopathy with masked IgG kappa deposits (MGMID) was recognized in 2011 and described by Larsen et al. in 2014 as a distinct glomerulopathy. Typical findings on light microscopy (LM) are mesangial hypercellularity, focal segmental glomerulosclerosis, and crescent formation, with interstitial fibrosis also noted in over half of the biopsies. Typical findings on electron microscopy are subepithelial and mesangial deposits, with occasional subepithelial humps and hinge-region deposits, and rare subendothelial deposits. On immunofluorescence with frozen tissue (IF-F), staining for C3 is common. Immunofluorescence on formalin-fixed, paraffin-embedded tissue (IF-P) is positive for IgG (always IgG1 when tested), kappa [1], and serum amyloid P (SAP) [2], and negative for phospholipase A2 receptor and thrombospondin type 1 domain containing 7A. Occasional weak (1+) positive staining for IgM and IgA is noted [1].

In an updated case series of 41 patients published by Larsen et al. in 2016, MGMID predominantly affected females (female-to-male ratio 3.6:1) under the age of 40 years and was associated with vague autoimmune phenomena, though rarely with well-defined autoimmune disease. These cases represented 0.1% of 42,711 kidney biopsies over five years. The most common clinical findings were hematuria (88%), proteinuria (nephrotic range in 35%), positive autoimmune serologies (55%), and elevated serum creatinine (29%). Of note, only one patient met criteria for systemic lupus erythematosus (SLE). Furthermore, no cases exhibited paraproteinemia [3].

## Case presentation

An 18-year-old female with recent influenza B infection, anemia, fatigue, and arthralgias presented to the rheumatology clinic after testing positive for ANA with a mildly elevated anti-dsDNA level. During her hospitalization for influenza, she was found to have a hemoglobin level of 6 g/dL and received three units of packed red blood cells. Colonoscopy and EGD ruled out bleeding. Hematologic workup revealed undetectable haptoglobin and a positive direct antiglobulin test for IgG and C3d, consistent with warm autoimmune hemolytic anemia.

The patient was referred to rheumatology, where she met criteria for SLE. At this visit, she had an elevated urine protein-to-creatinine ratio (UPCR) and normal serum creatinine. All lab values are summarized in Table 1.

**Table 1:** Pertinent laboratory values ANA, antinuclear antibody; anti-β2GP1 IgG, anti-beta-2 glycoprotein 1 IgG antibodies; anti-β2GP1 IgM, anti-beta-2 glycoprotein 1 IgM antibodies; anti-dsDNA, anti-double-stranded deoxyribonucleic acid antibodies; anti-La/SSB, anti-La/anti-Sjögren’s-syndrome-related antigen B antibodies; anti-Ro/SSA, anti-Ro/anti-Sjögren’s-syndrome-related antigen A antibodies; anti-Sm, anti-Smith antibodies; GP1 IgG units, glycoprotein 1 IgG units; GP1 IgM units, glycoprotein 1 IgM units; UPCR, urine protein-to-creatinine ratio

Lab	Value	Reference range
ANA titer	1:320	<1:80
Anti-dsDNA (IU/mL)	16	<10
Anti-Sm (U/mL)	1.2	<7
Anti-Ro/SSA (U/mL)	>240.0	<7
Anti-La/SSB (U/mL)	26	<7
Anti-β2GP1 IgM (GP1 IgM units)	88	<32
Anti-β2GP1 IgG (GP1 IgG units)	85	<20
Haptoglobin (mg/dL)	<30	40-240
UPCR (g/g)	2.2	<0.2
Creatinine (mg/dL)	0.85	0.6-1.60

She was referred to pediatric nephrology, and a kidney biopsy was performed. In the interim, she was started on high-dose prednisone and hydroxychloroquine. The biopsy showed MGMID without interstitial fibrosis or tubular damage. Of the 26 glomeruli sampled, two were globally sclerotic.

**Figure 1:**
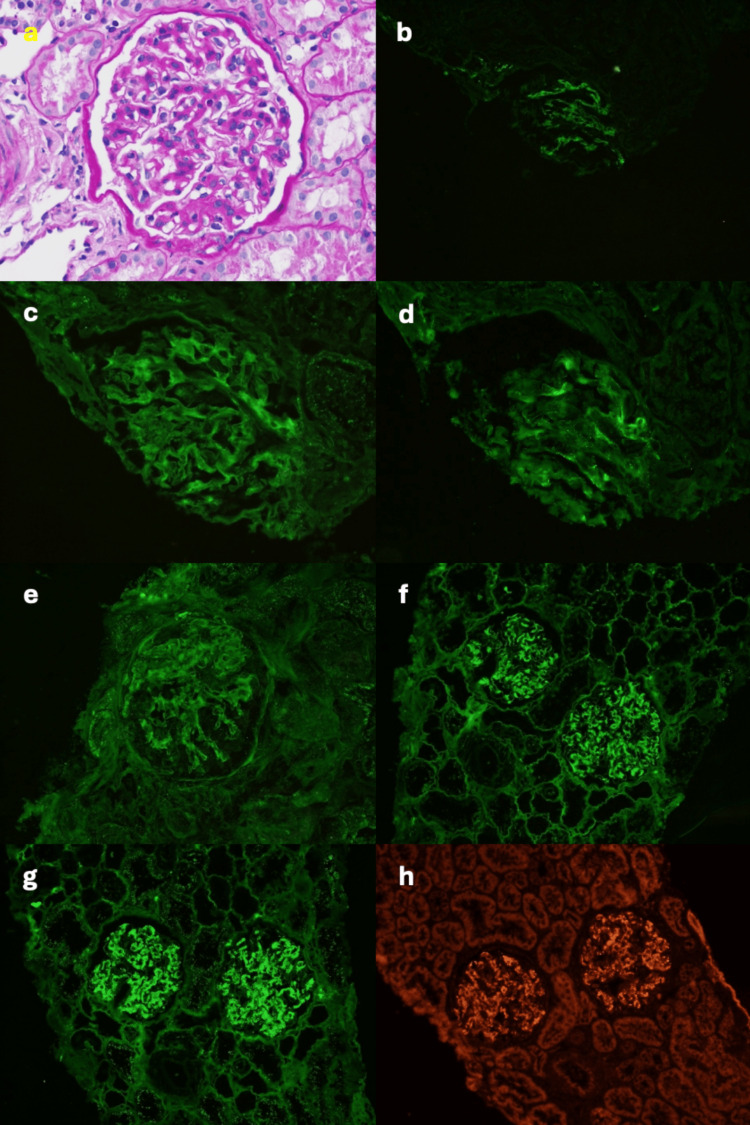
Representative kidney biopsy images (a) LM stained with hematoxylin and eosin showing diffuse mild mesangial matrix expansion. (b) IF-F showing 1-2+, granular mesangial C3 staining. (c) IF-F showing 1+, granular mesangial IgG staining. (d) IF-F showing 1-2+, granular mesangial IgM staining. (e) IF-F showing 2+, granular mesangial kappa staining. (f) IF-P showing 3+, granular mesangial and paramesangial IgG staining. (g) IF-P showing 3+, granular mesangial and paramesangial kappa staining. (h) IF-P showing 2+ SAP staining. C3, complement component 3; IF-F, immunofluorescence on fresh frozen tissue; IF-P, immunofluorescence on paraffin-embedded tissue; LM, light microscopy; SAP, serum amyloid P component.

Histopathologic images demonstrated weak mesangial staining for IgG and kappa on IF-F, with strong mesangial and paramesangial staining for IgG and kappa on IF-P, confirming the diagnosis of MGMID. SAP staining was strongly positive. Representative images from the biopsy are shown in Figure 1.

Following a multidisciplinary discussion, the patient was prescribed mycophenolate mofetil. At follow-up, she had not initiated the medication due to fear of side effects, though her UPCR had declined to 0.93 g/g on prednisone and hydroxychloroquine alone. She was then initiated on mycophenolate, and at six months, she was compliant and demonstrated further improvement with UPCR reduced to 0.30 g/g.

## Discussion

Renal biopsy interpretation relies on LM, IF-P, and EM. When frozen tissue is unavailable or diagnostic clarity is lacking, IF-P is a critical salvage technique. Initially used to detect conditions like light chain proximal tubulopathy [4], IF-P has since revealed previously unrecognized patterns of immune complex deposition.

Unlike previously described entities such as proliferative glomerulonephritis with monoclonal Ig deposits and monoclonal Ig deposition disease, MGMID is characterized by weak or absent IgG staining on routine IF-F, but has strong staining for IgG kappa “unmasked” via IF-P. While MGMID shares the predominant subepithelial deposits on EM commonly seen in membranous nephropathy (MN), MN typically stains strongly for IgG (especially IgG4 in the primary form) on IF-F. Infection-related glomerulonephritis (IRGN) also tends to have subepithelial deposits on EM, but usually shows IgA, IgM, and/or IgG staining on IF-F. Furthermore, levels of C3 are often low in IRGN but normal in MGMID. IRGN shows endocapillary proliferation on LM, unlike MGMID, and IRGN is temporally associated with infection. Yet another mimic is C3 glomerulonephritis (C3GN), which shares C3-predominant staining with minimal Ig deposition on IF-F. However, patients with C3GN usually have membranoproliferative glomerulonephritis (MPGN) on LM and low serum C3. Given these similarities, it is likely that MGMID was misclassified as atypical membranous glomerulopathy, IRGN, or C3GN before 2011 [1].

Since 2011, IF-P has uncovered additional instances of masked Ig deposits. In 2015, Larsen et al. reported 16 patients with MPGN with masked monotypic Ig deposits, often with paraproteinemia and low serum C3 level [5]. In 2020, DiFranza et al. described a case of IRGN with masked IgGk crystalline hump-like deposits in the setting of hypocomplementemia, endocapillary proliferation, and active infection [6]. Also in 2020, Nagahama et al. documented a patient with Sjögren’s syndrome who developed MN with masked polyclonal IgG deposits. These deposits were polyclonal, predominantly IgG4 (in contrast to the IgG1 pattern in MGMID), and negative for SAP staining [7]. Finally, IF-P has proven useful in differentiating type I from mixed cryoglobulinemia by enabling antigen detection otherwise hidden within crystalline immune deposits [4]. A comparative summary of MGMID and these related conditions is provided in Table 2.

**Table 2:** Comparison of MGMID, MN, C3GN, and IRGN ^‡^ All four diseases can have presentations varying from asymptomatic proteinuria and/or hematuria to nephrotic syndrome and/or elevated creatinine [1,8-10]. ANA, anti-nuclear antibody; ASO, anti-streptolysin O; C3GN, C3 glomerulonephritis; C3NeF, C3 nephritic factor (anti-C3bBb); C4NeF, C4 nephritic factor (anti-C4b2a); C5NeF, C5 nephritic factor (anti-C3bBbC3b); EM, electron microscopy; FSGS, focal segmental glomerulosclerosis; GBM, glomerular basement membrane; HBV, hepatitis B virus; HCV, hepatitis C virus; HIV, human immunodeficiency virus; IF-F, immunofluorescence with frozen tissue; IF-P, immunofixation with formalin-fixed, paraffin-embedded tissue; IE, infective endocarditis; IgG4RD, IgG4-related disease; IRGN, infection-related glomerulonephritis; LM, light microscopy; MesPGN, mesangioproliferative glomerulonephritis; MGIMD, membranous-like glomerulopathy with masked IgGκ deposits; MM, multiple myeloma; MN, membranous nephropathy; MPGN, membranoproliferative glomerulonephritis; NELL, neural epidermal growth factor-like 1 protein; PLA2R, phospholipase A2 receptor; PSGN, post-streptococcal glomerulonephritis; RA, rheumatoid arthritis; SLE, systemic lupus erythematosus; SAP, serum amyloid P; SjS, Sjögren’s syndrome; THSD7A, thrombospondin type-1 domain-containing 7A; WM, Waldenström’s macroglobulinemia

Characteristic	MGMID	MN	C3GN	IRGN
Age	<40 years	>40 years	<50 years	Children/young adults (PSGN), older adults (IgA-dominant form)
Sex (F:M)	3.6:1	1:2	1.7:1	Slight male predominance (IgA-dominant)
Associations	Vague autoimmune features, rarely SLE	SLE, SjS, RA, IgG4RD, HBV, HCV, HIV, syphilis, malignancy, drugs, heavy metals	Upper respiratory tract infection, MM, WM	*Streptococcus* *pyogenes*, *Staphylococcus aureus*, IE
Distinguishing clinical findings^‡^	None	Underlying systemic disease	Recent infection or paraprotein	Preceding or concurrent infection
Serum C3	Normal	Normal	Low	Low
Other labs	ANA+	PLA2R, NELL, or THSD7A Ab	C3NeF, C4NeF, or C5NeF	ASO or anti-DNAse B (PSGN)
LM	Mesangial hypercellularity, FSGS, crescent formation, interstitial fibrosis	Diffuse capillary and GBM thickening, with GBM spikes and pinholes	MPGN (most common), MesPGN, endocapillary proliferation	Endocapillary proliferation, rare scattered subendothelial deposits, occasional MesPGN, MPGN, and crescents
EM	Subepithelial deposits	Subepithelial deposits	Subepithelial deposits	Subepithelial deposits
IF-F	C3 +, weak or no IgG	C3 +, IgG + (esp. IgG4)	C3 +, weak or no IgG	C3 +, usually with IgA, IgM, and/or IgG +
IF-P	IgGk +, SAP +	N/A	N/A	N/A

We report a case of MGMID in an 18-year-old female with well-defined SLE, normal renal function, and sub-nephrotic proteinuria. To our knowledge, this is the second published case of MGMID in a patient with SLE.

Miura et al. previously reported a patient with SLE and MN without Ig deposition. IF-P was not performed in this case, suggesting this may be an undiagnosed case of MGMID [11].

SLE is a well-known cause of secondary MN [8]. However, the classic histologic findings in lupus nephritis include immune complex deposition that is readily visible on IF-F. In contrast, MGMID is characterized by “masked” IgG kappa deposits that are undetectable or weakly positive on IF-F, but strongly positive on IF-P. This suggests that MGMID may have been historically misclassified as atypical MN, IRGN, or C3GN prior to the widespread adoption of IF-P.

The presence of monoclonal staining in MGMID without paraproteinemia, especially in young females with autoimmune features, challenges traditional concepts of monoclonal gammopathy-related kidney disease. While most reported MGMID cases are associated with vague autoimmune serologies, they rarely meet full criteria for autoimmune diseases like SLE. Our case adds to the limited literature supporting an association between MGMID and defined autoimmunity.

Given its recent discovery and rarity, the pathogenesis of MGMID remains unclear. It is unknown why the IgG kappa deposits are “masked” on standard IF-F and unmasked by protease digestion in IF-P. Whether MGMID represents a unique disease entity or a subtype of MN or lupus nephritis is also uncertain. Similarly, its long-term clinical course and optimal treatment strategies are not well-defined. In the largest case series to date by Larsen et al., of the 27 patients for whom treatment was reported, the most common was a combination of corticosteroids and mycophenolate mofetil (26%), followed by no treatment (22%, with half of these not treated due to the presence of end-stage renal disease (ESRD) at presentation) and RAAS blockade alone (22%), and corticosteroids and calcineurin inhibitor (15%). Other immunosuppressive agents used include rituximab (4%), azathioprine (4%), hydroxychloroquine (4%), and combination therapy (4%). The rates of partial or full remission among those who received no treatment (and did not have ESRD), RAS blockade alone, and immunosuppression were 100%, 50%, and 60%, respectively [3]. These results suggested that immunosuppressive therapies were varied, and outcomes were inconsistent. Our patient experienced a steady decline in proteinuria following corticosteroids, hydroxychloroquine, and initiation of mycophenolate mofetil, suggesting this approach may be beneficial.

## Conclusions

This case highlights a rare presentation of MGMID in a patient with established SLE, emphasizing the diagnostic utility of IF-P in patients with unclear or discordant biopsy findings or atypical presentations of glomerulonephritis. We hope that this report and others like it enable physicians to learn about and more easily recognize MGMID. Much remains unclear about the link between MGMID and autoimmunity. Further research is needed to clarify the pathogenesis, autoimmune associations, and treatment responses in this emerging clinicopathological entity.
